# The three pillars of tomorrow: How Marketing 5.0 builds on Industry 5.0 and impacts Society 5.0?

**DOI:** 10.1016/j.heliyon.2024.e36543

**Published:** 2024-08-23

**Authors:** Mihalj Bakator, Dragan Ćoćkalo, Vesna Makitan, Sanja Stanisavljev, Milan Nikolić

**Affiliations:** University of Novi Sad, Technical Faculty “Mihajlo Pupin”, Đure Đakovića bb, 23000, Zrenjanin, Republic of Serbia

**Keywords:** Marketing 5.0, Industry 5.0, Society 5.0, AI, Technology, Competitiveness

## Abstract

In today's environment, the connections between Marketing 5.0, Industry 5.0, and Society 5.0 are gaining increasing attention. Governments and businesses are eager to explore how they can boost both economic competitiveness and societal well-being through strategic initiatives. It is important to ensure that technology adoption, ethical governance, and human capital development all align and are in-sync. This review dives into this challenge, aiming to create a theoretical model that provides significant insight on how Marketing 5.0 influences Society 5.0 through Industry 5.0. By analyzing a broad range of literature, the aim was to offer practical suggestions and guidelines for enhancing competitiveness and societal welfare. 48 studies were analyzed studies to discuss the complexities of the relationships between these three domains. The findings suggest actionable steps and strategies for both businesses and policymakers. Finally, the paper serves as a foundation for future research in this area, exploring the synergy between Marketing 5.0, Industry 5.0, and Society 5.0.

## 1 Introduction

The modern business environment has brought tremendous changes to the competitive relations between enterprises. The development and widespread adoption of information and communication technologies (ICTs) have had a substantial impact on social, political, and economic structures [1,2]. The fast evolution of production methods, driven by the concepts of Industry 4.0 and Industry 5.0, has transformed how businesses operate and significantly altered the socio-economic dynamics of countries [3]. The concept of Society 5.0 emerges as the solution to the challenges of optimized production and work allocation within the framework of Industry 5.0. It represents a futuristic vision where digital transformation and societal needs converge [4]. It is a human-centered society that integrates cyberspace and physical space. Society 5.0 is not just about technological advancement; it's a vision for a new societal model [5]. The concept emphasizes the use of advanced technologies like AI, IoT, and big data to solve societal problems, thereby transcending the conventional boundaries of Industry 4.0 [6,7]. Society 5.0 aligns closely with the Sustainable Development Goals (SDGs), envisioning a society that utilizes technology to develop sustainable and eco-friendly solutions, thereby effectively addressing environmental issues and resource management [8]. Moreover, besides the use of technology creating sustainable solutions, data and their availability in the open data form are seen as an asset for this, acting as both a resource and a tool for achieving Sustainable Development Goals (SDGs) and fostering smart living [9]. This aligns with the broader objectives of Society 5.0, which include sustainable development, enhanced public welfare, and tackling global challenges like aging populations and environmental issues [10]. While Society 5.0 is a broader societal vision, Industry 5.0 focuses more on the industrial aspect. Industry 5.0 is an evolution beyond Industry 4.0, emphasizing personalized and sustainable production [11]. This concept introduces a strong human touch to the technological advancements of Industry 4.0, balancing efficiency with social and environmental responsibility [12]. Information technology plays a crucial role in the transition to Industry 5.0. This evolution is marked by improved collaboration between humans and machines, striving to create a more sustainable, resilient, and personalized manufacturing process [13]. There is a significant intersection and co-evolution between Society 5.0 and Industry 5.0. Both visions are complementary and advocate for the integration of advanced technologies with human-centric approaches [12,14]. Society 5.0 provides the overarching framework for societal transformation, while Industry 5.0 outlines a roadmap for industrial evolution within this context [11]. Industry 5.0 introduces changes not only in manufacturing [15] but also in other sectors such as transportation and logistics [16], marketing [17], and communication [18]. Marketing 5.0, building on the broader Industry 5.0 concept, aims to maintain competitiveness and sustainability within Society 5.0 [19,20]. These three concepts are pivotal pillars of modern social, political, and economic dynamics. Marketing 5.0 is a complex framework that encompasses real-time analytics [[21], [22], [23]], customer value creation, support, user experience, segmentation [24], and green marketing [25]. Through customer feedback loops [26], businesses can focus on quality control and improvement [27]. Industry 5.0 facilitates on-demand production [12], which supports flexible scheduling [[28], [29]] and other factors complementary to Society 5.0. Together, Industry 5.0 and Marketing 5.0 enable enterprises to adapt to the evolving demands of Society 5.0 [30].

The existing literature extensively explores these three concepts individually, with only a few studies examining them collectively. Consequently, this systematic review aims to analyze the synergy among these concepts. The need for this review arises from the necessity to expand the current literature and establish a foundation for future research discussions. The objective is to develop a comprehensive theoretical model based on Marketing 5.0, Industry 5.0, and Society 5.0, exploring the complexity and interrelationships among these concepts.

The paper is organized into five main sections. First, key ideas and definitions are introduced. This is followed by a detailed description of the methodology. Next, the results from the qualitative analysis are discussed. The paper then presents the developed theoretical model. Recommendations for governments and enterprises are explored in the Discussion section. Finally, the paper concludes with final thoughts and suggestions for future research.

## 2 Methodology

### 2.1 Research questions

The systematic literature review was initiated to examine the domains of Industry 5.0, Marketing 5.0, and Society 5.0. The purpose of this review is to explore the interactions between these concepts, focusing on their interconnections and mutual influences. This investigation is crucial for understanding the strategies that governments and enterprises can employ to enhance competitiveness and societal well-being, ensuring strategic coherence among technological adoption, ethical governance, and human capital development. Additionally, the outcomes of this systematic literature review can provide a robust framework serving as a basis for future research. The review aims to address the following research questions.

• RQ1: What factors and concepts characterize Marketing 5.0, Industry 5.0 and Society 5.0?
• RQ2: Does Marketing 5.0 affect Society 5.0 through Industry 5.0?
• RQ3: How does Marketing 5.0 affect Society 5.0 through Industry 5.0?
• RQ4: What actions can governments and enterprises take to achieve and maintain competitiveness within these concepts?

### 2.2 Review protocol details

The systematic literature review was carried out in three main phases: planning the review, conducting the review, and reporting the results. Each phase consisted of several stages designed to ensure a rigorous approach to the review process. These stages were performed following best practices in the field of systematic literature reviews [31,32]. Given the multidisciplinary nature of the research topics, databases such as Scopus, Web of Science, IEEE Xplore, DOAJ, JSTOR, ScienceDirect, ERIC, and the KoBSON platform were selected. This diversified selection aimed to mitigate indexing biases and ensure a comprehensive capture of studies across fields like technology, marketing, industrial management, and social sciences. Google Scholar was also included as a supplementary engine to capture gray literature and extend the review's reach beyond traditional academic databases, providing a broader scope of insights and perspectives.

In the first stage of conducting the systematic review, after the preliminary database selection, the focus was on the actual identification of research within those databases. This involved executing a detailed and structured search strategy developed to capture literature relevant to the research questions surrounding Marketing 5.0, Industry 5.0, and Society 5.0. The search strategy was operationalized through a combination of keywords and search queries, aligning with the thematic and conceptual framework of the review.

The **keywords** and search queries that were used in the database search process are presented in Table 1.

**Table 1:** Keywords and search queries.

Keywords that were added to the search queries	
marketing 5.0; Society 5.0; Industry 5.0; AI; cobots; IoT; sustainability; e-government	
Search queries	
1. renewable energy adoption case studies	20. development of autonomous vehicles
2. data privacy in digital education	21. impact of self-driving cars on urban mobility
3. cybersecurity trends	22. marketing 5.0 and Society 5.0
4. security challenges in technology	23. marketing 5.0 and Industry 5.0
5. digital marketing in e-commerce	24. Industry 5.0 and Society 5.0
6. trends in online marketing	25. customer value
7. consumer behavior in digital shopping	26. society wellbeing
8. technology in smart city development	27. AI-driven technologies
9. urban traffic management technology	28. advanced manufacturing
10. urban planning	29. environmental regulation
11. telemedicine advancements	30. customer value
12. digital technology in healthcare	31. marketing communication
13. applications in medical services	32. innovations
14. blockchain applications in enterprise	33. digital transformation in manufacturing
15. latest innovations in renewable energy	34. customer engagement
16. emerging trends in green energy solutions	35. impact of chatbots on branding
17. social media analytics for business	36. customer interaction studies
18. strategies using social media data	37. green technology in business
19. trends in social media marketing analytics	

Keywords were added to the search queries. Keywords were the “base” while queries were used in combination with the keywords.

The search engines returned the same results regardless of whether singular or plural forms of words were used, as the text within the documents was also searched. Thus, the choice between singular and plural forms did not impact the search results. This stage involved interacting with the selected databases and applying the search strategy across each platform. The process was iterative, with initial search results reviewed for relevance and scope, leading to refinements in the search queries and keywords as needed. The goal was to include relevant literature that would help address the research questions. Key elements of this stage include.

• Implementation of search strategy: The application of the predefined search queries and keywords across the selected databases to gather a preliminary set of studies.
• Initial screening: A **first screening** of the search results to exclude duplicate records and remove incomplete or corrupt files that where inaccessible. Microsoft Excel was used for initial labeling while the Systweak Duplicate Files Fixer was used to remove duplicates.
• Documentation of search process: Keeping a detailed record of the search strategy implementation, including search terms used, and databases searched.

Furthermore, utilizing the previously selected databases, a **second screening** process was conducted where the search strategy, incorporating specific keywords and queries related to Marketing 5.0, Industry 5.0, and Society 5.0 were taken into consideration regarding the titles and abstracts. This selection process was important in narrowing down the large body of literature to a focused collection of primary studies that are directly relevant and contribute valuable insights to the research questions. The collaborative effort of the review team, supported by the diversity of databases, ensured a balanced and comprehensive selection of primary studies.

Following the selection of primary studies, the next step was to assess the methodological quality and credibility of these studies to ensure that the review's findings are based on reliable and valid research. This was the **third screening** process where five reviewers assessed each report. **Details about the reviewers**.

• *Reviewer 1:* Specializes in marketing management and Internet marketing with a focus on their application in economic and industrial settings. Interests are on exploring the intersection of technology and society, particularly how emerging technologies can influence societal developments in Society 5.0.
• *Reviewer 2:* Extensive experience in entrepreneurship, industrial management and the integration of technological innovations in business processes within Society 5.0.
• *Reviewer 3:* The expertise primarily involves ICT application in business and marketing strategies, with a particular emphasis on digital marketing and its impacts on consumer behavior and business outcomes.
• *Reviewer 4:* Specializes in technological advancement and manufacturing with the focus on Industry 4.0 and Industry 5.0. Interests also include how new sustainable technologies can be adapted to the changing landscape of digital and global markets.
• *Reviewer 5:* Expertise is in the domain of strategic management and business decision making. Focuses on analyzing economic and business trends to provide strategic recommendations for organizations navigating complex and dynamic markets.

The reports were assessed for eligibility through the inclusion and exclusion criteria. The **exclusion criteria were**.

- Lack of credibility: this refers to the absence of methodological rigor, the lack of transparent presentation of results, limitations, and the lack of information if the paper was peer-reviewed (This was identified as a journal that publishes scientific papers with a similar model as books where peer-reviews are not practiced). The number of removed records was 11.
- Lack of peer-review: peer review is a key factor in establishing validity by experts in the field. The lack of peer-review indicates inadequate evaluation of the study before publication. Therefore, 2 studies were excluded in the identification step.
- Lack of relevancy: this refers to how the subject of the article aligns with the goal of the review. A relevant article contributes directly to the understanding, development, or discussion of the subject matter at hand. It should have a clear connection to the key themes, questions, or objectives outlined in the review. In this current study, if the reviewer noticed in the abstract or Introduction that the study was not relevant, it was excluded from further assessment. Based on the lack of relevancy, 23 records were removed.
- Lack of methodological quality: high methodological quality refers to a study that clearly defines its research questions, uses appropriate and well-justified methods to collect and analyze data, and thoroughly reports its procedures and findings. Such a study would enable others to evaluate the reliability and validity of its results. There were 5 studies removed due to the lack of methodological quality.
- Inappropriate research design: refers to the lack of adequately aligned research goals with the objectives or questions of the study. Inappropriate design undermines the validity and reliability of interpreted results. For this review, articles that didn't have a clear methodological framework (sample, data collection, assessment tools) were flagged with inappropriate research design. The lack of appropriate research design is not often present in peer-reviewed articles; However, there were 9 studies that were excluded.
- Low quality of presentation: implies to the quality of how the research sections are presented. Academic standards regarding this are flexible across domain. However, studies that didn't include Introduction, Methodology, and Results with a Discussion were excluded from the final set of items included in the review. Additionally, if the results were partially presented or there was no Discussion, the paper was not included in the final set of items. In total, four papers were excluded due to low quality of presentation.
- Not generalizable: this term refers to results of the study or the context that are not applicable or interpreted to contexts outside the specific conditions of the conducted study. This can be due to a highly specific sample, unique experimental conditions, or the study addressing a specific one-time, non-reoccurring event. In the screening process, there were se 7 studies that were excluded due to them not being generalizable.
- Unsuitable outcome measures: this term refers to the selection of measures or indicators in a study that are not appropriate to assess the intended outcomes or objectives. If a study used a subjective measure for a construct that requires an objective measurement, then the study was not considered in the final set of items.

The **inclusion criteria were** the opposite of the aforementioned exclusion criteria.

- Credibility: This pertains to the presence of methodological rigor, transparent presentation of results, limitations, and confirmation of peer review status. Only studies demonstrating a high level of credibility, as evidenced by clear and comprehensive reporting of their methodology, findings, and limitations, will be considered for inclusion. The inclusion of credible records is crucial to ensure the reliability and validity of the review findings.
- Peer-reviewed: Peer review is instrumental in validating a study's findings by experts in the respective field. Only studies that have undergone a rigorous peer-review process will be included. This criterion ensures that each study considered has been evaluated and endorsed by qualified reviewers, establishing a foundation of validity and reliability.
- Relevancy: Relevance to the review's objectives is a primary criterion for inclusion. Studies must directly contribute to the understanding, development, or discussion of the review's subject matter. They must exhibit a clear and direct connection to the key themes, questions, or objectives of the review. This relevance should be evident from the study's abstract or introduction, indicating a direct alignment with the core topics of interest. Records that do not meet this criterion of relevancy will be excluded.
- Methodological quality: High methodological quality is essential for inclusion. Studies must clearly define their research questions and employ appropriate, well-justified methods for data collection and analysis. They should thoroughly report their procedures and findings, enabling an evaluation of the results' reliability and validity. The inclusion of studies with high methodological quality is vital for ensuring that the review's findings are based on robust and reliable evidence.

After the inclusion and exclusion criteria were applied to the search results, studies that did not meet the quality and relevance standards were excluded, while 48 studies were included in the review. The **quality assessment** served as a guide to further refine the selection, excluding studies that did not meet the predefined quality benchmarks. The reviewed studies were different in settings, time, and populations. This diversity contributes in understanding the findings. Multiple reviewers assessed the same study. This included using a template for determining the eligibility of the study. Additionally, **coefficients** were given by each reviewer. Triangulation didn't create a double/triple amount of extracted review results, but rather it helped to achieve a more balanced and reliable understanding of the topic.

The reviewers have developed a template for a “Record assessment card”. This card was filled out by each reviewer after analyzing a paper. The “Record assessment card” template is given in Table 2.

**Table 2:** Record assessment card template.

RECORD ASSESSMENT CARD	
ID in Excel file:	PEER-REVIEW: YES NO
YEAR OF PUBLICAITON:	SOURCE:
(write OK beside the reviewer number)	EXCLUDE (write step and reviewer number beside reason)
Step 1 - Identification	Duplicate:
R1: R2: R3: R4: R5:	Lack of credibility:
Step 2: Screening	Lack of peer-review:
R1: R2: R3: R4: R5:	Lack of relevancy:
Step 3: INCLUDE	Lack of methodological quality:
R1: R2: R3: R4: R5:	Inappropriate research design:
	Low quality of presentation:
	Not generalizable:
	Unsuitable outcome measures:
IF INCLUDED based on your expertise give a coefficient from 1 to 10 as weight of significance. 1 being less significant, and 10 being more significant* The weights are then calculated by dividing the coefficient by 50.	R1: R2: R3: R4: R5:

An example of a filled out Record assessment card is presented on Fig. 1.

**Fig. 1:**
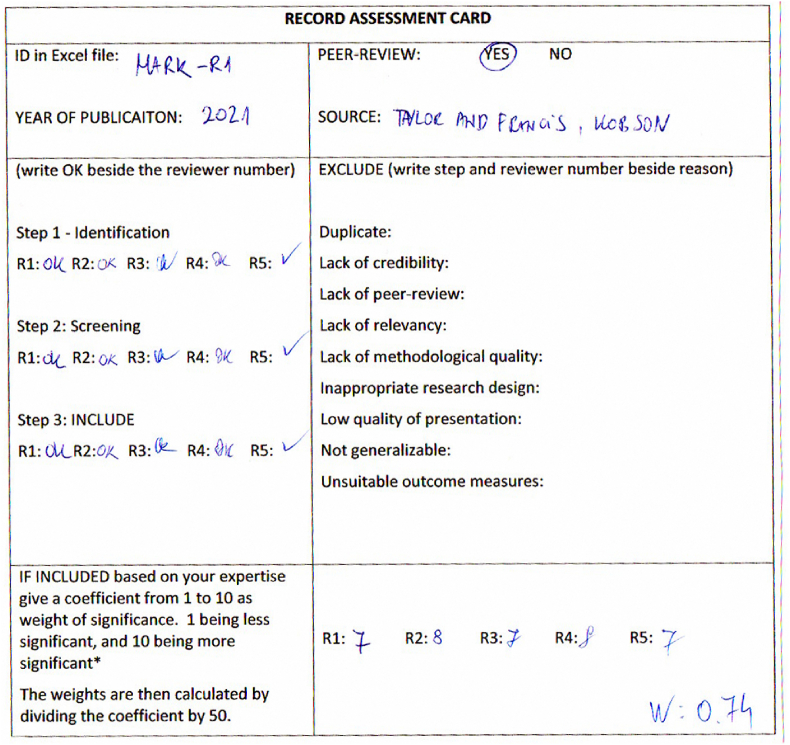
Record assessment card (filled).

The obtained **weights (W)** within the record assessment cards were not statistically analyzed, but they were interpreted in a qualitative manner in the final synthesis. The qualitative analysis results of each assessed study were not written on the record assessment cards. Instead, the text was written in a collaborative Word document. Explicitness in interpretation was assessed through the development of a theoretical model where each study presents a model element. The weight coefficients are presented alongside the developed model.

Furthermore, the studies that passed the **third screening process** underwent data extraction and synthesis process. Information relevant to the review's objectives was systematically extracted from each study, including key findings. The synthesis of data, leveraging the comprehensive and varied database selection, facilitated an analysis that integrated insights across different disciplines and methodologies. As the results of each study were qualitative in nature, a data meta-analysis was not conducted.

The majority of the articles reviewed were in English; however, other languages were also considered as long as they could be accurately translated. Priority was given to recently published articles, but studies from the recent past were also analyzed to assess their continued relevance. The most recent publications were from 2023, while the oldest were from 2017, with most publications appearing in the last five years.

This stage culminated in the development of a theoretical model that integrates the interrelations and dynamics between Marketing 5.0, Industry 5.0, and Society 5.0. Additionally, backups were created on various cloud platforms, such as Google Drive, Dropbox, and in-house systems, to mitigate the risk of data loss. Without backups, reviewers would have to retrieve records online again, which is time-consuming. Initially, Excel was the primary tool, but concerns about potential loss or corruption of Excel files led to the consideration of paper formats as semi-backups.

Microsoft Visio was used to construct the model, while Semantic Scholar was utilized for contextual analysis and exploration of research topics. To ensure the reliability of the findings, sensitivity analysis was conducted by removing studies to see if the overall conclusions changed. Criteria for exclusion included studies with significant biases, those not meeting pre-set methodological standards, and outliers with inaccurate methodologies. Comparing adjusted outcomes with the original findings helped assess the stability of the results and the influence of specific studies on the overall conclusions, determining whether the synthesis was overly dependent on a few studies or remained consistent across various designs and settings.

Literature searches were expanded to include gray literature to mitigate publication bias. Sources for gray literature included the World Economic Forum, Keidanren policies, handbooks published by IGI Global, and the United Nations Development Programme. This approach ensured a more accurate and comprehensive synthesis of evidence, grounding the conclusions of the systematic review in a balanced representation of available research.The assessment of confidence of findings in the literature was an integral part of the systematic review process. This involved a critical evaluation of the evidence's quality, consistency, and relevance.

The results of the qualitative assessment were used to develop a theoretical model and a development scenario diagram. The development process of the model began with the identification of the need to explore the interrelations between Marketing 5.0, Industry 5.0, and Society 5.0. Based on the insights gathered from the literature review, the researchers formulated multiple initial theoretical models. These models aimed to visually and conceptually represent the interactions and impacts of Marketing 5.0 on Society 5.0 through the mediation of Industry 5.0. The initial models were refined through multiple discussions within the research team. They were designed to be scientifically accessible and with visual representations and explanations. The relations between the model elements included the corresponding labels of an analyzed study.

Finally, suggestion and guidelines for improving the competitiveness of countries and enterprises and for improving society wellbeing were to be developed. The development of suggestions and guidelines was directly informed by the results of the systematic literature review. This review provided a comprehensive understanding of the existing knowledge and gaps in the fields of Marketing 5.0, Industry 5.0, and Society 5.0. Key insights and recurrent themes from the systematic review were synthesized to identify potential strategies and actions that could address the observed gaps and leverage identified opportunities within the three domains. The research team engaged in multiple deliberation sessions to discuss and refine these insights into practical suggestions and actionable guidelines. Each suggestion was scrutinized for its feasibility, relevance, and potential impact. Consensus on the final set of suggestions and guidelines was achieved through structured discussions. The process involved aligning on the objectives, scope, and expected outcomes of the proposed actions.

The procedure stopped when the resulting set of suggestions and guidelines was considered sufficient. This sufficiency was defined by the coverage of key areas identified during the literature review and the consensus among research team members that the recommendations were comprehensive, actionable, and aligned with the latest research and industry practices.

### 2.3 PRISMA protocol

**Fig. 2:**
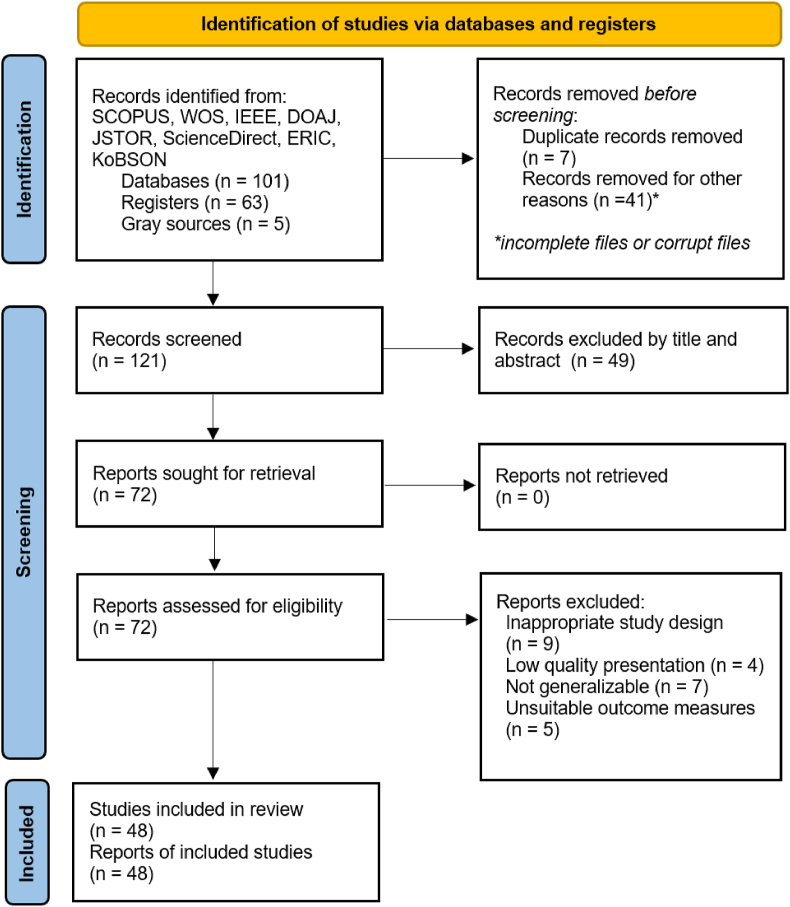
Preferred Reporting Items for Systematic Reviews and Meta-Analysis (PRISMA) protocol flow diagram.

The study used the well-established and reliable Preferred Reporting Items for Systematic Reviews and Meta-Analysis (PRISMA) approach [33,34]. Fig. 2 shows the flow diagram.

## 3 Results

The results of the qualitative analysis are presented through a generalized approach in Table 3. (a more detailed analysis is presented in Appendix Table A1). Every reference was labeled (MARK-R1, IND-R1, SOC-R1, etc.). The MARK label refers to Marketing 5.0, the IND label refers to Industry 5.0, and the SOC label refers to the Society 5.0 concept. The labels are used for tracking the analyzed study and how is it noted within the theoretical model.

**Table 3:** Qualitative results.

Label	Study objective/contribution/findings	Key point
**MARK-R1**:	The study introduces real-time analytics in Marketing 5.0 to enhance customer engagement through personalized experiences [35].	real-time customer analytics
**MARK-R2**:	The findings advocate for the integration of predictive modeling to forecast consumer behavior for improved ROI and engagement [36].	predictive consumer behavior modeling
**MARK-R3**:	The paper highlights the effectiveness of customer segmentation in Marketing 5.0 using soft computing for increased satisfaction [37].	data-driven customer segmentation
**MARK-R4**:	AI-driven recommendations to personalize customer suggestions, boosting marketing communication is noted [38].	AI-powered recommendations
**MARK-R5**:	The study emphasizes the role of technology in improving customer experience and brand identification [39].	enhanced customer experience
**MARK-R6**:	Automated customer support systems, noting their impact on satisfaction and potential downsides if not well implemented is noted [40].	automated customer support
**MARK-R7**:	The evolution of customer feedback loops into real-time, data-driven tools for marketing strategy enhancement is explained [41].	real-time feedback analysis
**MARK-R8**:	The importance of digital channels in marketing for reaching audiences and personalized engagement is discussed [42].	digital marketing channels
**MARK-R9**:	Mobile app effectiveness in interactive marketing for personalized customer approaches is analyzed [43].	mobile marketing engagement
**MARK-R10**:	Marketing eco-friendly products for higher satisfaction and green consumer loyalty is described [44].	eco-friendly marketing
**MARK-R11**:	The value of social responsibility messaging in marketing for stakeholder value creation is showed [45].	social responsibility messaging
**MARK-R12**:	The integration of online-offline channels for a seamless customer journey is discussed [46].	online-offline integration
**MARK-R13**:	The importance of a unified customer journey across all touchpoints for marketing success is noted [47].	unified customer journey
**MARK-R14**:	The need for content aligned with brand values to positively impact customer emotions was found to be important [48].	brand-aligned content
**MARK-R15**:	Content marketing with user-generated content on brand attitude, showing no significant difference was analyzed [49].	user-generated content impact
**MARK-R16**:	The importance of brand transparency in advertising for trust and loyalty is noted [50].	brand transparency
**MARK-R17**:	The role of fair trade certifications in marketing for ethical and sustainability goals was addressed [51].	ethical and sustainable marketing
**IND-R1**:	IoT sensors' role in enhancing operational efficiency and productivity in manufacturing was highlighted [52].	IoT in manufacturing efficiency
**IND-R2**:	Quality 5.0's proactive approach to quality control, balancing technology with human creativity was introduced [53].	personalized quality control
**IND-R3**:	Collaborative robots' contribution to a synergistic work environment combining human creativity and robotic efficiency was described [54].	human-robot collaboration
**IND-R4**:	AR training's impact on skill development and data's indirect effect on Society 5.0 through decision-making and automated systems was explored [55].	AR for skill development
**IND-R5**:	Just-in-time manufacturing's evolution with data analytics for reduced inventory costs was noted [56].	just-in-time manufacturing innovation
**IND-R6**:	Blockchain technology's role in creating transparent and sustainable supply chains was outlined [57].	blockchain for supply chain transparency
**IND-R7**:	Renewable energy sources in reducing carbon footprints and enhancing operational efficiency was highlighted [58].	renewable energy in industry
**IND-R8**:	Waste minimization strategies for material conservation and process optimization was noted [59].	waste minimization strategies
**IND-R9**:	Digital simulation models for anticipating operational bottlenecks and improving efficiency was analyzed [60].	digital operation simulations
**IND-R10**:	Challenges in adopting predictive maintenance solutions in industrial settings were discussed [61].	predictive maintenance challenges
**IND-R11**:	The important role of intrusion detection systems in securing digital infrastructure was highlighted [62].	digital infrastructure security
**IND-R12**:	The importance of secure data transmission using encryption for operational integrity was noted [63].	secure data transmission
**IND-R13**:	On-demand production's flexibility and customization benefits driven by real-time analytics was discussed [64].	on-demand production flexibility
**IND-R14**:	Local sourcing to enhance supply chain resilience and sustainability was analyzed [29].	local sourcing strategy
**IND-R15**:	The necessity of adhering to environmental regulations for sustainable industrial operations was covered [65].	environmental regulation compliance
**IND-R16**:	Safety standards integration technology for risk minimization was noted [66].	safety standards evolution
**SOC-R1**:	Traffic surveillance advancements for improved congestion monitoring and multimodal transportation analysis was described [67].	advanced traffic surveillance
**SOC-R2**:	Public WiFi's role in supporting smart city innovations and community engagement was highlighted [68].	public WiFi for smart cities
**SOC-R3**:	Telemedicine's transformation of healthcare through accessible, precise remote diagnoses and monitoring was detailed [69].	telemedicine accessibility
**SOC-R4**:	AI-assisted diagnosis in healthcare for early detection and personalized treatment was examined [70].	AI in healthcare diagnostics
**SOC-R5**:	e-learning platforms' contribution to personalized, accessible education [71].	personalized e-learning
**SOC-R6**:	Lifelong learning initiatives for adapting to evolving professional and societal demands were noted [72].	lifelong learning programs
**SOC-R7**:	Green buildings' impact on sustainable urban development through technology integration were analyzed [73].	green building technologies
**SOC-R8**:	The efficiency and cost-competitiveness of solar, wind, and hydro technologies in advancing sustainable energy solutions was highlighted [74].	renewable energy adoption
**SOC-R9**:	The role of income equality measures, like progressive taxation and universal basic income, in mitigating wealth gaps was discussed [75].	income equality initiatives
**SOC-R10**:	The transformative impact of remote work technologies on work-life balance and environmental sustainability was explored [76].	remote work evolution
**SOC-R11**:	The benefits of flexible scheduling in enhancing productivity and supporting a diverse workforce were analyzed [77].	flexible work schedules
**SOC-R12**:	E-government services improving administrative efficiency and citizen engagement through digital platforms was noted [8].	e-government efficiency
**SOC-R13**:	Transparent policymaking to foster an inclusive approach to governance and enhance public engagement was discussed [78].	transparent policymaking
**SOC-R14**:	The challenge of balancing data privacy with innovation, emphasizing the need for strong encryption and ethical data collection were addressed [79].	data privacy challenges
**SOC-R15**:	The importance of ethical AI in ensuring fairness and inclusivity in technology deployment and addressing biases was highlighted [80].	ethical AI implementation

Additionally, based on the reviewer expertise a coefficient from 1 to 10 as weight of significance (1 being less significant, and 10 being more significant) was given to each article. The weights are then calculated by dividing the sum of five coefficients by 50 (five coefficients were given as there were five reviewers). The calculated weight coefficients from each assessment card (W value) are presented in Table 4. These weight coefficients contribute to interpreting the model elements.

**Table 4:** Weight coefficients.

**Label**	**Weight coefficient**	**Label**	**Weight coefficient**	**Label**	**Weight coefficient**
MARK-R1	0.74	IND-R1	0.84	SOC-R1	0.78
MARK-R2	0.70	IND-R2	0.78	SOC-R2	0.80
MARK-R3	0.66	IND-R3	0.66	SOC-R3	0.76
MARK-R4	0.72	IND-R4	0.60	SOC-R4	0.56
MARK-R5	0.76	IND-R5	0.60	SOC-R5	0.54
MARK-R6	0.58	IND-R6	0.54	SOC-R6	0.88
MARK-R7	0.60	IND-R4	0.64	SOC-R7	0.70
MARK-R8	0.54	IND-R8	0.62	SOC-R8	0.50
MARK-R9	0.50	IND-R9	0.70	SOC-R9	0.62
MARK-R10	0.56	IND-R10	0.72	SOC-R10	0.68
MARK-R11	0.62	IND-R11	0.78	SOC-R11	0.66
MARK-R12	0.88	IND-R12	0.70	SOC-R12	0.72
MARK-R13	0.58	IND-R13	0.60	SOC-R13	0.58
MARK-R14	0.62	IND-R14	0.52	SOC-R14	0.60
MARK-R15	0.66	IND-R15	0.56	SOC-R15	0.62
MARK-R16	0.68	IND-R16	0.54		
MARK-R17	0.82				

**Fig. 3:**
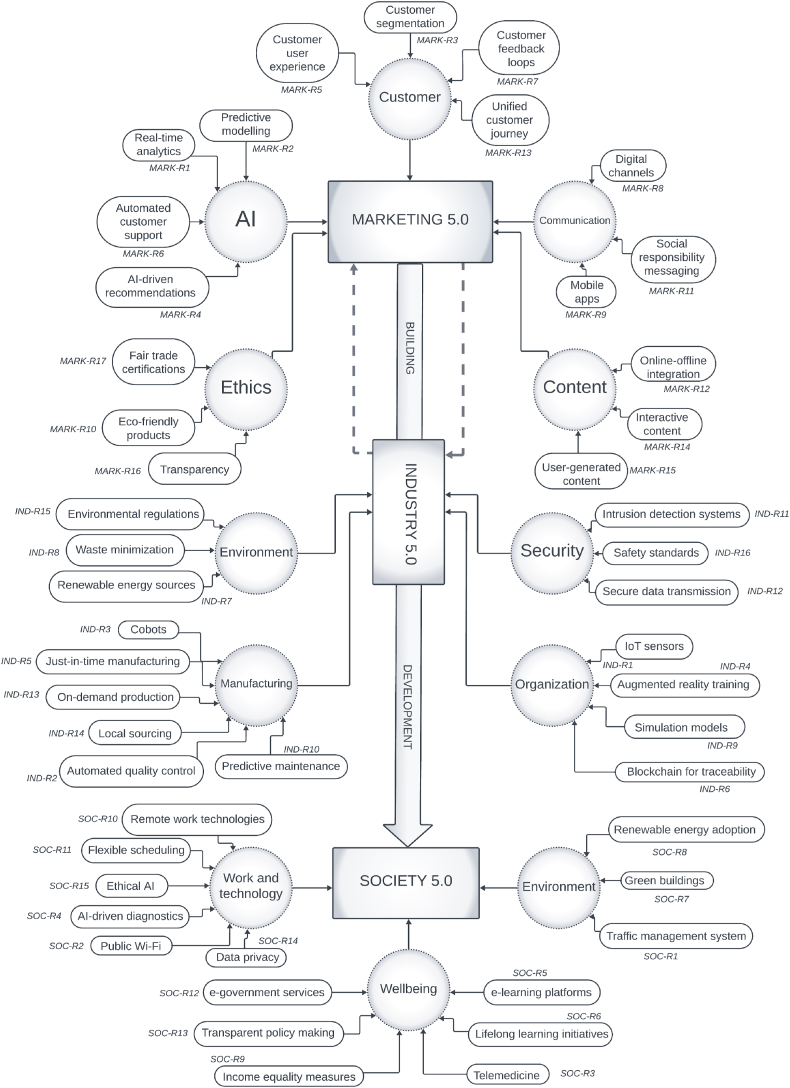
Theoretical model.

The labels are then presented in the developed theoretical model. The labels refer to the domain to which the article corresponds. Based on the synthesis of the literature review, key elements and constructs that were significant to the field of study were identified. These elements represented the core concepts that the theoretical model aims to explain. The connections/relationships between the elements were established based on the relationships observed or inferred from the literature. For each connection, the corresponding label (or code) from the analysis phase indicated the empirical or theoretical basis for noting this relationship, linking back to specific findings or themes from the literature review. The model is presented on Fig. 3.

**Fig. 4:**
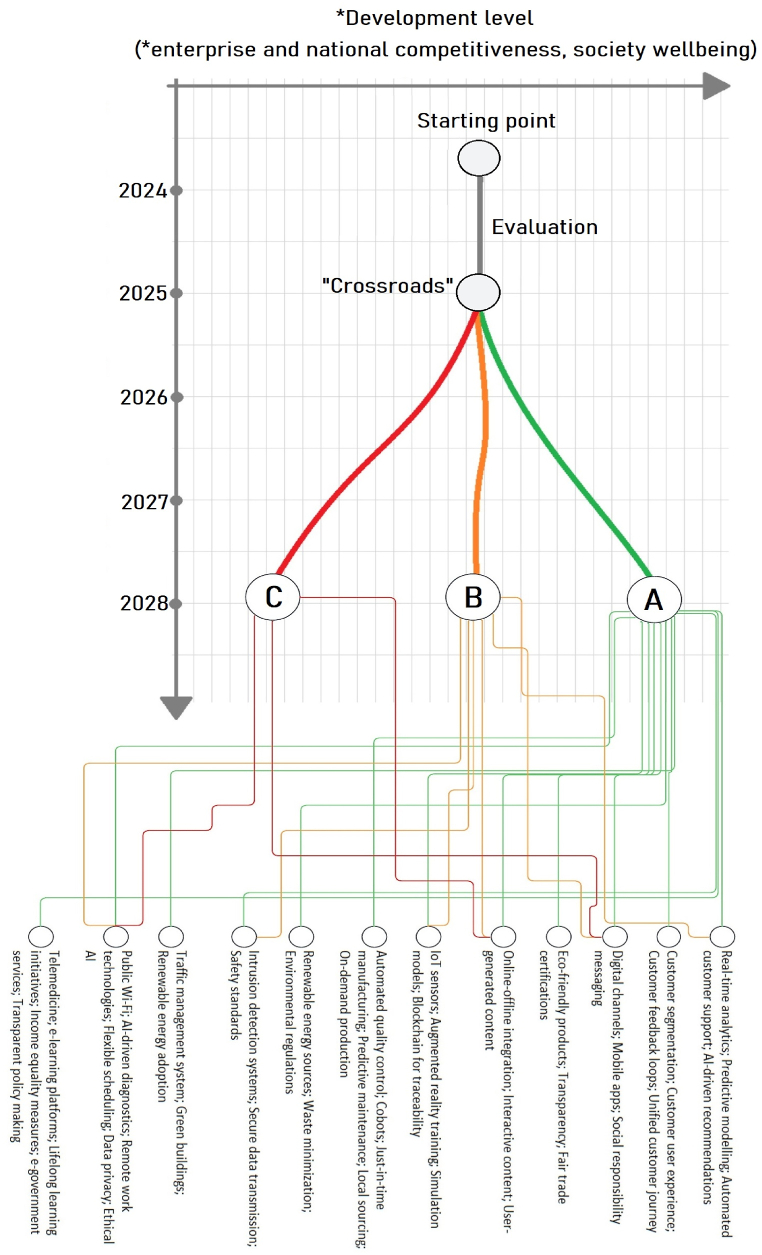
Theoretical map of potential development scenarios.

It is evident, based on the model, that Marketing 5.0 builds on Industry 5.0, each with its own individual sub-elements, and as such, goes towards Society 5.0 which is affected by the synergy of the previous two concepts. Labels are beside each element and correspond to the previously noted results of the qualitative analysis. Next, a theoretical map of potential scenarios of development in the domain of Society 5.0 is presented on Fig. 4.

Depending on the extent to which technological and other solutions are implemented, different outcomes or scenarios can be expected. These scenarios were defined through approximations based on expert opinions, knowledge, and literature analysis.

The most favorable scenario, Scenario A, envisions a future where the full spectrum of available technologies is harnessed to create a society that is sustainable, efficient, and human-centric. In this scenario, the integration of AI and IoT facilitates the creation of smart cities that optimize resource use and enhance citizens' lives through personalized services. Smart robotics enable flexible manufacturing processes that can quickly adapt to changing demands while ensuring sustainability. The widespread adoption of renewable energy sources is critical for reducing carbon footprints and combating climate change. This scenario is underpinned by the belief that technological advancements, when fully implemented, have the potential to address the most pressing societal challenges.

Scenario B can be described as a "status quo" outcome, where some solutions are implemented, but no major changes occur in the domain of renewable energy sources. This scenario reflects a more cautious approach, where incremental changes are made without fully committing to the transformative potential of new technologies. The adoption of AI and IoT is limited to applications that do not significantly disrupt existing economic and social structures. Renewable energy technologies are adopted only in areas where they are most cost-effective or politically feasible. This scenario suggests a world where progress is made, but the transformative potential of a fully integrated Society 5.0 is not realized due to economic, political, or societal resistances.

The least favorable outcome, Scenario C, describes a situation where minimal advancement occurs due to various barriers such as economic constraints, lack of political will, or societal resistance to change. Technologies like AI and IoT are used in a limited capacity, primarily in sectors where they do not challenge existing power structures or economic models. Renewable energy adoption is slow and fails to significantly replace fossil fuels. This scenario highlights the risks of inaction and the potential consequences of failing to embrace the technologies and strategies necessary for a sustainable and prosperous future.

The starting point differs from country to country and from enterprise to enterprise. In the evaluation phase, the necessary changes are examined, and potential technological solutions and strategies are discussed. This choice reflects an analysis of progress across sectors, recognizing that advancements may not proceed at an equal pace. The manuscript clarifies that decision-making and implementation efforts span across national, regional, and international levels, acknowledging the complexity and variability of energy policy and technological adoption. The reference to a "crossroad" in 2025 signifies a critical juncture for prioritizing actions that align with the envisioned pathways towards a sustainable future, based on a consensus among reviewers and an analysis of the current trajectory of technological and societal advancements.

Research indicates a significant increase in the attention towards AI, IoT, and smart cities post-2015, highlighting an upward trajectory in the development and application of these technologies [81]. This trend suggests a movement towards the maturation of these technologies by 2025, where they are expected to play a central role in the transformation of urban spaces into smart cities. These smart cities are characterized by the integration of IoT, AI, and big data analytics to enhance sustainability, efficiency, and the overall well-being of citizens, with a strong emphasis on implementing renewable energy sources and eco-friendly initiatives [81,82].

The societal readiness for embracing digital and sustainable technologies is also evident in the growing number of smart city initiatives worldwide. The smart city paradigm has evolved to include a broad range of technologies and strategies aimed at improving urban life through increased efficiency, reduced resource consumption, and improved quality of life for citizens. This readiness is a reflection of the public's increasing awareness and acceptance of digital solutions and sustainable practices as essential components of future urban development [81].

Addressing environmental urgencies, such as climate change and resource depletion, necessitates a shift towards more sustainable urban planning and development strategies. The concept of sustainable smart cities, which integrates data-driven technologies and solutions with green technologies, is becoming increasingly popular as a way to address these challenges. These cities aim to become environmentally sustainable by optimizing energy efficiency, improving air quality, and enhancing waste management through the use of advanced technologies [82].

The convergence of AI, IoT, and big data technologies is a key factor in the evolution of environmentally sustainable smart cities. These technologies enable the collection, analysis, and application of data in ways that significantly improve the sustainability and livability of urban environments. The literature underscores the importance of these technologies in driving the thematic evolution of sustainable urbanism and highlights the need for further research to better understand their integration and impact [83].

It is important to note that the concepts and technologies of Marketing 5.0 and Industry 5.0 are viewed within the context of Society 5.0. The years are taken as an approximation provided by the consensus between the reviewers and based on studies regarding Marketing 5.0 [84,85], Society 5.0 [86,87], sustainable development [88], and technological advancement in Industry 5.0 [89]. The theoretical map serves as an idea for interpretation rather than a definitive course of development paths and timeframes. 2030 is the half-way point for Sustainable development Goals on a global level. However, discussing potential scenarios on itself can be challenging as data is coming in constantly regarding the different aspects of development on a national, regional, and international levels. Therefore, we concluded that it is best to put 2025 as a starting point from which we observe how the development of different aspects will play out and which scenario will come to fruition. In practice, it is not possible to accurately predict if some scenarios will play out completely as noted, but rather a combination of realized goals and a mix of scenarios. The Figure's main goal is discussion, where the framework is provided vie this article, and the scenarios, crossroads are "flexible" for further evaluation by the reader.

Society 5.0, Industry 5.0, and Marketing 5.0 represent interconnected frameworks shaping modern socio-economic landscapes. Society 5.0 envisages a human-centric society empowered by advanced technologies, bridging digital and physical realms to enhance quality of life and sustainability [90]. This concept integrates technologies like the Internet of Things (IoT), artificial intelligence (AI), and big data into daily life to address social challenges such as aging populations, urbanization, and environmental sustainability. The aim is to create a super-smart society where digital innovations solve complex societal issues, improve healthcare, and enhance overall well-being [90].

Industry 5.0 extends this vision to manufacturing and production, emphasizing collaborative human-machine systems that promote customization and efficiency [91,92]. In contrast to Industry 4.0, which focuses on automation and data exchange, Industry 5.0 highlights the collaboration between humans and machines. This approach ensures workers are central to production processes, using AI and robotics to enhance human capabilities and creativity. The concept integrates artificial intelligence and digitalization to optimize production systems in a transformative manner [93]. For instance, robots working alongside humans on assembly lines enhance precision and allow greater product customization to meet individual consumer needs. This combination of human ingenuity and machine efficiency boosts productivity and fosters innovation and job satisfaction [[91], [92], [93]].

Marketing 5.0 complements these advancements by focusing on innovative approaches that merge technological innovation with profound customer insights, fostering personalized and ethical marketing practices [[94], [95], [96]]. This approach uses AI, data analytics, and digital tools to understand and anticipate consumer behavior, delivering highly personalized marketing strategies. The goal is to build customer loyalty and increase market share through strategic engagement and personalized experiences [95]. Marketing 5.0 also emphasizes ethical considerations, ensuring that marketing practices are effective, responsible, and aligned with societal values. This includes transparent data usage, respect for consumer privacy, and creating authentic brand-consumer relationships [[94], [95], [96]].

Together, these frameworks represent a comprehensive evolution towards integrating technology not only for economic advancement but also for societal welfare, ensuring a harmonious balance between human needs and technological progress [96,97]. This holistic approach demonstrates the potential of advanced technologies to drive sustainable development, enhance human capabilities, and create inclusive growth. By fostering collaboration between various sectors and prioritizing human-centric values, Society 5.0, Industry 5.0, and Marketing 5.0 collectively pave the way for a future where technological advancements contribute to a more equitable, efficient, and sustainable world [96,97].

## 4 Discussion

### 4.1 Main concepts “pillars” of the model

The three pillars - Marketing 5.0, Industry 5.0, and Society 5.0 - are deeply integrated, often in subtle ways that are not immediately apparent. Artificial intelligence (AI) is a foundational technology that is revolutionizing all three industries. AI enables highly personalized customer experiences in Marketing 5.0, while it improves automation and predictive maintenance in Industry 5.0. AI is used in Society 5.0 for important public services such as traffic management and healthcare diagnostics. AI advancements in these sectors create a symbiotic relationship in which innovations in one drive progress in the other. AI algorithms developed for Industry 5.0, for example, could potentially be adapted to predict and prevent infrastructure failures in Society 5.0, thereby contributing to public safety.

Next, another important cross-pillar component is adherence to ethical and environmental standards. In Marketing 5.0, ethical considerations such as fair trade can compel Industry 5.0 to adopt more responsible practices, pushing Society 5.0 towards sustainability and equality. Industries are being forced to adapt as consumers become more aware and demanding of ethical business practices and sustainability. This sets off a chain reaction in which ethical consumer choices can lead to fundamental shifts in societal values and norms.

The “glue” that holds these pillars together is data. In Marketing 5.0, data analytics provide insights into consumer behavior, which then informs production methods and quality control in Industry 5.0. Data is used for traffic management, healthcare, and governance in Society 5.0, effectively making data management and data privacy a collective responsibility that spans these pillars. In essence, the data privacy and security measures in one pillar set the standard for the others.

Marketing 5.0's advanced analytics enable an in-depth understanding of consumer preferences, which shapes production methods in Industry 5.0. For example, if there is a consumer trend toward eco-friendly products, Industry 5.0 could shift to more sustainable manufacturing methods, such as the use of renewable energy. These adaptations can help achieve Society 5.0's overarching goals, such as lowering carbon emissions and accelerating the adoption of renewable energy.

The insights and data-driven approaches of Marketing 5.0 directly influence Industry 5.0. Understanding consumer preferences and behaviors allows industries to tailor their production processes to create more personalized and desirable products. This consumer insight is vital for industries to stay relevant and competitive in a rapidly changing market. The advancements in Industry 5.0, particularly in creating personalized and sustainable products, contribute significantly to the realization of Society 5.0. When industries focus on sustainable practices and cater to the unique needs of individuals, it leads to a more sustainable, inclusive, and personalized society. There's a cyclical influence among these three concepts. The societal changes and demands in Society 5.0 can influence the direction of Marketing 5.0, which in turn shapes industrial practices in Industry 5.0. This cycle continues as each evolution in one area sparks advancement in the others. Furthermore, the efficiencies gained from predictive modeling in Marketing 5.0 can be transferred to Industry 5.0, resulting in more economically sustainable production cycles. These advantages can then have an impact on Society 5.0 by potentially lowering consumer costs, improving living standards, and contributing to income equality.

### 4.2 Sub-elements of the model

As for the sub-elements of the three pillars, it can be noted that real-time analytics and predictive modeling guide customer segmentation in Marketing 5.0, influencing the design of AI-driven recommendations. These technologies aren't stand-alone features; they're part of an ecosystem that includes automated customer support and feedback loops, which refine predictive models and improve the user experience.

Additionally, these data-driven practices integrate seamlessly with digital channels, mobile apps, and both online and offline solutions. They also lay the groundwork for socially responsible initiatives such as eco-friendly products and fair trade certifications, in response to consumer insights into ethical consumption preferences.

Internet of Things (IoT) sensors and automated quality control systems are the cornerstones upon which predictive maintenance and collaborative robot (cobot) deployment rely in Industry 5.0. They are inextricably linked to just-in-time manufacturing processes, which are further optimized by simulation models. Renewable energy sources and waste reduction are not optional extras; they are key sub-elements that align with the growing societal emphasis on sustainability. They also cross paths with other regulatory frameworks, such as environmental regulations and safety standards. Add in the use of blockchain for traceability and secure data transmission, and you have an interconnected landscape with societal implications, particularly in areas such as public trust and data privacy.

Looking at Society 5.0, it can be seen that traffic management systems and public Wi-Fi are built on the same foundational technologies that support telemedicine and e-learning platforms. These digital infrastructures also support flexible work arrangements and lifelong learning programs, which are societal changes that Industry 5.0 advancements have accelerated. The emphasis on ethical AI and data privacy in Society 5.0 intersects with similar concerns in Marketing 5.0 and Industry 5.0, creating a common ground for ethical practices and regulatory compliance across all three pillars.

Examining how Marketing 5.0 affects Society 5.0 via Industry 5.0 reveals some intriguing synergies. If Marketing 5.0 detects an increase in consumer demand for environmentally friendly products, Industry 5.0 may be encouraged to adopt renewable energy sources and waste minimization techniques. This alignment would not only meet market demand but would also contribute to societal goals such as the adoption of renewable energy and waste reduction. Similarly, the data gathered and analyzed in Marketing 5.0 may provide useful insights for public services in Society 5.0. Consumer mobility data, for example, could be invaluable for optimizing traffic management systems, resulting in a more efficient and sustainable urban environment.

Marketing 5.0, Industry 5.0, and Society 5.0 should be viewed as interconnected components of a larger, evolving ecosystem rather than as distinct entities. Marketing 5.0 has an impact on consumer and industrial behavior, which in turn has an impact on societal norms, practices, and even public policy. This comprehensive viewpoint reveals how intertwined these pillars truly are, with each influencing the other in a web of complex, yet enormously impactful, relationships. Next, the research questions are addressed.

RQ1. What factors and concepts characterize Marketing 5.0, Industry 5.0, and Society 5.0?

Key factors in Marketing 5.0 include real-time analytics, predictive modeling, AI-driven recommendations, and a greater emphasis on social responsibility, such as eco-friendly products and fair trade. These components work together to create a unified, personalized, and ethically responsible customer experience. Automation and data-driven efficiency characterize Industry 5.0, with a strong emphasis on sustainability and ethical operations. This includes Internet of Things (IoT) sensors, collaborative robots (cobots), predictive maintenance, renewable energy sources, and waste reduction. The goal here is to integrate human creativity with technological innovation to create a more sustainable and adaptive industrial landscape. Traffic management systems, public Wi-Fi, telemedicine, e-learning platforms, and green buildings are examples of societal changes driven by technological innovation and ethical imperatives. These are intended to improve people's lives, promote sustainability, and provide more equitable access to resources and opportunities.

RQ2. Does Marketing 5.0 affect Society 5.0 through Industry 5.0?

Based on the analyzed literature it can be noted that there is relationship present between these concepts. Marketing 5.0, which uses advanced technology and data to understand customers, deeply influences how Industry 5.0 operates. This means that factories and companies are now making products and providing services that are not just high-tech but also really in-sync with what consumers want and need. As a result, this change in how things are made has a big impact on society as a whole. Society 5.0, which is all about using technology to make life better for society, benefits from this. Therefore, when marketing gets smarter and more focused on the individual, it leads to smarter manufacturing, and in turn, this helps create a society that's more advanced and more focused on people's needs. Thus, the trajectory from Marketing 5.0 to Society 5.0, through Industry 5.0, illustrates a complex but integral relationship where advancements in marketing and industry collectively foster a society that aligns technological progress with human well-being.

RQ3. How does Marketing 5.0 affect Society 5.0 through Industry 5.0?

Marketing 5.0 has an impact on Society 5.0 via its influence on Industry 5.0, acting as a catalyst for broader societal change. For example, if Marketing 5.0 detects a consumer trend toward environmentally friendly products, Industry 5.0 can adapt by implementing renewable energy sources and waste minimization techniques. These industrial shifts then contribute to societal goals such as reduced carbon emissions and increased use of renewable energy.

Data analytics and predictive modeling techniques used in Marketing 5.0 can also be used to streamline production in Industry 5.0, with potential benefits such as lower costs being passed on to consumers, indirectly affecting living standards in Society 5.0.

The fourth research question is answered in the next section of the paper.

### 4.3 Suggestions and guidelines

RQ4. What actions can governments and enterprises take to achieve and maintain competitiveness within these three concepts?

Based on the qualitative analysis, theoretical model, map of development, and discussion, the following suggestions and guidelines for improving the competitiveness of countries and enterprises and for improving society wellbeing, are noted.

• Governments should invest in R&D which can aid in the acceleration of innovation. Specialized AI, Internet of Things (IoT), and blockchain research centers focused on addressing the specific challenges of Industry 5.0, Marketing 5.0, and Society 5.0 could be established.
• Governments and industry leaders can collaborate closely to co-create solutions such as Smart City technologies. Partnerships, for example, can be formed to develop AI-based traffic management systems that integrate real-time data from various sources, including enterprise-developed consumer mobile apps. Regulatory bodies can be established specifically to oversee data privacy, ethical AI, and sustainable practices. These organizations can conduct regular audits, publish compliance reports, and serve as arbiters in the event of a dispute.
• Governments and educational institutions can work together to incorporate cutting-edge courses in data analytics, automation, and ethical business practices into existing curricula. To upskill the existing workforce, public workshops and online courses can be offered.
• Governments could lead international initiatives to establish global standards for ethical AI, such as algorithm transparency, data privacy, and bias prevention in machine learning models.
• AI can be used for more than just customer segmentation and predictive modeling. It can also be used for internal decision-making processes, resource allocation, and even automating routine administrative tasks, freeing up human resources for more complex tasks.
• Enterprises should think about implementing closed-loop supply chains that reduce waste by designing products that can be recycled or repurposed. Sustainable initiatives should be communicated to consumers in a transparent manner via eco-labels and digital channels.
• Real-time analytics should be used not only to tailor products but also to provide predictive customer service, offering solutions before the consumer is even aware of a problem.
• Blockchain technology could also be used to provide real-time wage and working condition reports, significantly improving an enterprise's social responsibility standing. This level of radical transparency has the potential to distinguish a brand in a crowded marketplace.
• Cybersecurity measures must include regular training for employees to identify phishing scams, ransomware, and other cyber threats, in addition to protecting customer and enterprise data.
• In collaboration with tech enterprises, specialized bootcamps could be developed to provide hands-on training in emerging technologies. AI-powered assessment tools can be used to track employee performance in these programs.
• A council comprised of representatives from both sectors could be formed to constantly update and implement ethical and sustainable standards, such as the right to digital privacy and fair wages.
• For research purposes, joint databases can be created that are fully anonymized to protect privacy. This data set has the potential to provide important insights into public health, environmental conditions, and employment trends.
• Town hall meetings, online forums, and social media polls can all be used to actively engage communities. The information gathered can then be used to inform public policy and business strategies.
• Digital inclusion initiatives should aim for digital fluency rather than simply providing access. This could include educational programs that teach critical digital skills to citizens of all ages, allowing them to effectively interact with advanced technologies.

Governments and enterprises can contribute to the competitiveness and well-being goals inherent in the concepts of Marketing 5.0, Industry 5.0, and Society 5.0 by taking these actions and deploying these strategies in a deeply interconnected manner.

### 4.4 Limitations

The main limitations and potential biases of the are.

• There are certainly studies that didn't fit the specified criteria, potentially omitting relevant studies. This can potentially lead to skewed interpretations.
• The review protocol was not registered at the organization where the review was conducted, but it was thoroughly planned out between the authors.
• Studies with positive results are more likely to be published compared to those with negative or inconclusive results and this can lead to overrepresentation of positive findings. Unpublished papers or non-peer reviewed papers were not taken into consideration. This can lead to potential biases that should be addressed in the future.
• The quality of the studies affect the quality of the review results. Biased studies can affect the overall credibility of the interpretations.
• Variability in study designs, populations, interventions, and outcomes across the reviewed studies can make it challenging to draw general conclusions. Therefore, the diversity of the results is highlighted in the results section.
• Narrowly focused articles can miss the broader implications, while broad studies might lack depth in specific areas. This is why the defined protocol has a clearly defined scope and objectives. However, the limitation can't be fully eliminated, especially in this type of review.
• Recent studies may receive excessive emphasis, while older but still relevant studies are overlooked, potentially leading to time lag bias.

Researcher beliefs, roles, and ideas can significantly affect how the findings are interpreted. These beliefs, ideas, and roles are.

• Expertise and specialization is a situation where a researcher's specific area of expertise can shape their perspective. Someone deeply specialized in a narrow field might have a different view on a paper's novelty or importance compared to a more generalist researcher.
• Researchers can have methodological preferences for certain research methods or approaches. However, this refers to how the findings are interpreted and not exclusion. Thus, the preferences didn't affect the results.
• Personal beliefs, whether conscious or unconscious, can influence a reviewer's objectivity. This can include biases related to the author's institution, nationality, or gender.
• Some reviewers might be more open to innovative, risky, or interdisciplinary research, while others might prefer more traditional, established lines of inquiry.
• Researchers might be influenced by their own academic goals, such as maintaining a particular scientific narrative, supporting their own research agenda, or competing for grants and recognition. However, the above noted structured protocol was created to avoid this.

The reviewers were following the defined protocols and criteria with the goal to avoid the influence of the above noted roles, beliefs, and ideas.

### 4.5 Implications

The integration of Marketing 5.0 with Industry 5.0 is shown to drive significant improvements in economic competitiveness. Through leveraging AI, IoT, and big data, companies can create more personalized and efficient production processes, leading to higher customer satisfaction and, ultimately, economic growth. The systematic review highlights the role of technological adoption in stimulating innovation and productivity. Industry 5.0, characterized by a human-centric approach to manufacturing, combines the strengths of humans and machines, fostering an environment conducive to innovation and efficiency.

The alignment of Marketing 5.0 and Industry 5.0 with the principles of Society 5.0 promotes sustainable economic growth. The emergence of Society 5.0 necessitates the development of new policies and regulations that support ethical governance, data privacy, and security. Governments are required to create a regulatory environment that fosters the growth of Industry 5.0 and Marketing 5.0 while ensuring that technological advancements benefit society as a whole.

The interconnectedness of Marketing 5.0, Industry 5.0, and Society 5.0 underscores the need for global collaboration and the establishment of international standards, especially in areas like digital trade, cybersecurity, and technological innovation. The review article discusses how advancements in these three pillars can lead to improved social well-being and quality of life. The integration of ethical considerations into marketing and industrial practices is emphasized as a way to ensure sustainability and social responsibility. This includes fair trade, reducing carbon footprints, and promoting renewable energy sources.

## 5 Conclusion

The interdependence of Marketing 5.0, Industry 5.0, and Society 5.0 provides a complex yet promising framework for the future. The key to realizing their full potential lies in strategic alignment between governments and enterprises. Both sectors have the potential to boost economic competitiveness while significantly contributing to societal well-being. By focusing on cutting-edge technological adoption, ethical governance, and human capital development, they can co-create a future that is not only economically prosperous but also socially equitable and environmentally sustainable.

Several critical areas merit further investigation in future research. One pressing area is the ethical implications of AI, which is becoming increasingly pervasive in all aspects of life. Another important area is the creation of standardized sustainability metrics that can be universally applied across all three pillars—Marketing 5.0, Industry 5.0, and Society 5.0. This standardization could greatly simplify both compliance and regulation. Additionally, understanding the nuances of consumer behavior in an age of hyper-personalized, AI-driven marketing can provide valuable insights. Future research could also examine the growing digital divide, considering how rapid technological advances may exacerbate social and economic inequalities. Case studies of successful public-private partnerships can serve as models for multi-sector collaboration, and research into workforce adaptability to technological changes can offer insights into designing effective retraining programs.

In summary, numerous research opportunities exist that can provide deeper insights and actionable strategies, enhancing our understanding of the synergistic possibilities between Marketing 5.0, Industry 5.0, and Society 5.0.

## Ethics statement

Informed consent was not required for this study because there were no human nor animal subjects participating in experiments.

## Data availability

Has data associated with your study been deposited into a publicly available repository?: No.

Has data associated with your study been deposited into a publicly available repository?": Data included in article/supp. material/referenced in article.

## Funding details

No funding was received for this research.

## Disclosure statement

There are no conflicts of interest.

## CRediT authorship contribution statement

**Mihalj Bakator:** Writing – original draft, Visualization, Formal analysis, Data curation. **Dragan Ćoćkalo:** Writing – review & editing, Supervision, Methodology, Investigation, Conceptualization. **Vesna Makitan:** Writing – review & editing, Validation, Project administration, Conceptualization. **Sanja Stanisavljev:** Writing – review & editing, Resources, Investigation, Conceptualization. **Milan Nikolić:** Writing – review & editing, Resources, Methodology, Conceptualization.

## Declaration of generative AI and AI-assisted technologies in the writing process statement

During the preparation of this work the author(s) used QUILLBOT text editing in order to INCREASE FLUENCY of the manuscript. After using this tool/service, the author(s) reviewed and edited the content as needed and take(s) full responsibility for the content of the publication.

## Declaration of competing interest

The authors declare that they have no known competing financial interests or personal relationships that could have appeared to influence the work reported in this paper.

## Figures and Tables

**Table A1:** Qualitative results (expanded)

Label	Study assessment result
**MARK-R1**:	Marketing 5.0 represents the next frontier in customer engagement, focusing on leveraging cutting-edge technologies like Artificial Intelligence (AI), Internet of Things (IoT), and real-time analytics to deliver highly personalized experiences. In this paradigm, real-time analytics play a pivotal role in understanding customer behavior, preferences, and interactions almost instantaneously. Enterprises can monitor consumer metrics in real-time, allowing for more agile and precise decision-making. New trends in marketing development provide real-time analytics for customer value and for evaluating customer behavior. This can further increase customer satisfaction [35].
**MARK-R2**:	Predictive modeling uses advanced analytics, machine learning, and AI to forecast consumer behavior. Going beyond traditional methods, it interprets complex variables like real-time sensor data and social media activity to create highly personalized experiences. This data-driven approach allows enterprises to optimize ROI while enhancing customer engagement. Marketing researchers should integrate predictive modeling into their strategies, traditional cognitive and methodological procedures should not be completely abandoned [36].
**MARK-R3**:	Customer segmentation is the practice of dividing an enterprise's target market into distinct categories based on various traits like demographics, buying behavior, and psychographic characteristics. Customer segmentation through data analytics is an integral part of Marketing 5.0. Soft computing can significantly increase the response rate of customers and customer satisfaction [37].
**MARK-R4**:	AI-driven recommendations leverage machine learning algorithms to analyze customer data, from browsing history to past purchases, in real-time. This technology enables enterprises to offer highly personalized suggestions that resonate with individual needs and preferences. Integrated marketing communication can be enhanced with advanced information technologies, including AI-driven automation and recommendations. This approach can strengthen customer relationships and positively affect marketing goals [38].
**MARK-R5**:	Customer experience now hinges on a seamless integration of technology and human touch. Advances in AI, machine learning, and data analytics have equipped enterprises with the tools to understand and anticipate customer needs like never before. Marketing activities that contribute to user experience can help the customer identify with the brand, thus increasing future potential of customer engagement [39].
**MARK-R6**:	Automated customer support systems, powered by AI and machine learning, are transforming the way enterprises interact with their customers. These systems can also integrate with CRM platforms to provide personalized solutions, thus enhancing customer satisfaction. Automated customer support can be effective for increasing customer satisfaction. However, if the implemented solutions are not sophisticated enough, then it can heavily affect customer satisfaction in a negative way [40].
**MARK-R7**:	Customer feedback loops have evolved into real-time, data-driven mechanisms that capture and analyze user interactions across various channels. Enterprises use sophisticated analytics tools to continuously monitor customer sentiment, behavior, and needs. For an effective marketing strategy in the modern business environment, information analytics capabilities and applications should be tied to customer expectations. This can further lead to success of the digital business strategy [41].
**MARK-R8**:	Digital channels like social media, mobile apps, and e-commerce platforms have become essential touchpoints in today's customer journey. These platforms enable enterprises to reach audiences at scale while also offering granular targeting options for personalized interactions. In the context of green marketing, digital marketing communication channels should take into consideration SEO optimization, increasing web-page loading speeds, failure rate optimization, and remarketing activities in order to be successful [42].
**MARK-R9**:	Mobile apps serve as a vital conduit between enterprises and customers, offering a platform for personalized, on-the-go interactions. Leveraging real-time analytics and machine learning, these apps can deliver tailored content, promotions, and features to individual users. Mobile apps are a big part of interactive marketing strategies. The use of mobile apps in modern marketing campaigns is particularly effective in developing personalized approaches to customers. The result is an interactive process that is stronger compared to traditional approaches [43].
**MARK-R10**:	Eco-friendly products resonate strongly with today's increasingly conscientious consumers. Enterprises leverage advanced analytics to understand the specific sustainability concerns of various customer segments, then tailor their marketing messages accordingly. Developing marketing campaigns around eco-friendly products and services can lead to higher customer satisfaction and the development of green-movement-based customer loyalty [44].
**MARK-R11**:	Communicating social responsibility was found to have a significant potential to create value for stakeholders. Enterprises should consider using social responsibility messaging to create economic value and attract new customers [45].
**MARK-R12**:	Online-offline integration has emerged as a cornerstone of customer-centric strategies, blurring the lines between digital and physical touchpoints. Advanced analytics and Internet of Things (IoT) technologies enable a seamless transition between online browsing and in-store experiences, personalizing interactions across both realms [46].
**MARK-R13**:	The unified customer journey is a cohesive, cross-channel experience that caters to individual preferences and behaviors at each touchpoint. This 360-degree view allows for real-time adaptation and targeting, ensuring that messages and offerings remain relevant and engaging throughout the customer lifecycle. Customer journey success is largely affected by how well effectively the enterprise's online and offline communication channels [47].
**MARK-R14**:	An enterprise has to create content that is aligned with brand values and that is socially compelling to customers. It is necessary for the content to have a positive impact on the customers' feelings [48].
**MARK-R15**:	User-generated content (UGC), such as customer reviews, photos, and social media posts, has become a valuable asset in building brand authenticity and trust. Different content types result in differences in brand attitude. Serial mediators are conceptual and attitudinal persuasion knowledge. There was no discernible difference between content marketing and user-generated content [49].
**MARK-R16**:	Transparency has become a key differentiator in earning consumer trust and loyalty. This openness allows consumers to make informed choices, reinforcing a brand's credibility and reliability. In advertising, signaling brand transparency is beneficial in driving brand integrity. Perceived consumer empowerment mediates this connection. The results are consistent regardless of product complexity, design or signal source [50].
**MARK-R17**:	Fair trade certifications serve as a powerful tool to signal a brand's commitment to ethical sourcing, environmental sustainability, and social justice. Sustainability in marketing is a growing trend, and numerous other principles arise from it as ethical concerns grow. Often, marketing activities can contradict sustainability goals, as the goal of marketing is to sell as many products as possible, while sustainability concepts aim at reusability, recycling, and a circular economy approach with optimized exploitation of resources. However, based on an awareness of the reality of active green or ethical values regarding specific purchases, sustainability activities in marketing, as a behavior of green consumers, produce significant results [51].
**IND-R1**:	Internet of Things (IoT) sensors are revolutionizing the way enterprises operate by enabling real-time data collection and analytics. These sensors monitor various aspects such as temperature, pressure, and machinery health, leading to proactive maintenance and reduced downtime. Multiple manufacturing organizations have integrated the Internet of Things (IoT) into their production chain because this technology is the primary source of digitalization in a manufacturing facility. It boosts data assembly, operation productivity, communication efficiency, and overall production performance. The Internet of Things (IoT) can also help with better and more effective warehouse automation. It improves product delivery efficiency through calculating the shortest routes and also minimizes the time spent on inventory management [52].
**IND-R2**:	Quality 5.0 encompasses the integration of cutting-edge technologies like artificial intelligence and big data into quality control processes, placing a stronger emphasis on personalization, sustainability, and ethical manufacturing. It could also highlight a human-centric approach to automation, balancing technological advancements with human skills and creativity. It seeks to overcome the constraints of traditional quality control systems, which are often reactive; it takes a proactive approach to defect avoidance and process optimization [53].
**IND-R3**:	Collaborative robots (cobots), are designed to work alongside humans, providing a synergistic environment that combines human creativity with robotic efficiency. Unlike traditional industrial robots, cobots are equipped with sensors and machine learning algorithms that allow them to understand and adapt to human behavior [54].
**IND-R4**:	Augmented reality (AR) training programs elevate the learning experience and provide an immersive, interactive environment for skill development. Data powers Society 5.0 in two ways. Data indirectly affects social progress through influencing human decision-making. Data helps people make decisions that transform society and advance Society 5.0. For instance, transparent government data is essential to a sustainable economy. Second, data affects society through custom automated systems and processes. Streamlined picking mechanisms that use the Artificial Internet of Things (AIoT) link e-commerce platforms with Artificial Internet of Things (AIoT) systems and robots that meet consumer needs, speed up and simplify services for online retailers, and automated delivery systems [55].
**IND-R5**:	Just-in-time manufacturing has evolved to seamlessly integrate data analytics and smart technologies, enabling a more agile and responsive production line. This approach minimizes inventory costs by producing goods only as needed, based on real-time demand forecasts [56].
**IND-R6**:	Blockchain technology offers unparalleled traceability and transparency in supply chains. It creates immutable, decentralized records of each transaction or movement of goods. The concept of Industry 5.0 has given rise to a new perspective on sustainable supply chains (SSCs), emphasizing the importance of establishing a transparent and dependable network that can be consistently supervised and managed through stakeholder collaboration and the use of advanced, intelligent technology. The primary goal of Sustainable Supply Chains (SSCs) is to meet predetermined economic, social, and environmental performance benchmarks. The use of blockchain technology has the potential to greatly improve the dependability, effectiveness, and security of data shared among supply chain participants [57].
**IND-R7**:	Adoption of renewable energy sources like solar, wind, and hydropower is transforming industrial operations. These sources reduce carbon footprints and operational costs. These cleaner energy options are integrated through smart grids and real-time monitoring systems, enabling not just sustainability but also greater efficiency and reliability [58].
**IND-R8**:	Waste minimization strategies, including recycling and material recovery, have become integral components of modern industrial operations. Advanced sensors and analytics tools monitor resource usage in real-time, identifying opportunities for waste reduction and process optimization. These insights empower enterprises to adapt production cycles, conserve materials, and manage by-products more effectively [59].
**IND-R9**:	Digital simulation models enable enterprises to virtually replicate various aspects of their operations, from production lines to supply chain logistics. Organizations can test different scenarios, thus anticipating bottlenecks, optimizing workflows, and improving overall efficiency [60].
**IND-R10**:	Predictive Maintenance (PdM) solutions assist decision-makers in anticipating equipment health and strategically scheduling maintenance actions. However, implementing these solutions in industrial settings continues to be difficult. Previous research has consistently found that decision-makers are often hesitant to incorporate data-driven, system-generated recommendations into their operational protocols [61].
**IND-R11**:	Intrusion detection systems (IDS) serve as a critical line of defense in safeguarding an organization's digital infrastructure. These systems monitor network traffic and system activities for suspicious patterns or unauthorized actions, triggering alerts for immediate response. With the rise of interconnected devices and data flows, the role of IDS has become increasingly vital in maintaining operational integrity and safeguarding sensitive information [62].
**IND-R12**:	Secure data transmission is paramount in an era where interconnected systems generate and exchange vast amounts of sensitive information. Utilizing encryption protocols and secure communication channels, organizations can safeguard data integrity while it moves between devices, networks, and cloud-based platforms. Advanced techniques like end-to-end encryption ensure that data is only accessible to authorized users, adding an extra layer of security [63].
**IND-R13**:	On-demand production has emerged as a powerful approach to manufacturing, prioritizing flexibility and customer-centricity. This model also enables a higher degree of customization, allowing consumers to have products tailored to their specific requirements. The real-time analytics provide actionable insights into consumer preferences, further fine-tuning production processes [64].
**IND-R14**:	Local sourcing is gaining traction as a strategy to enhance supply chain resilience and sustainability. Enterprises can reduce transportation costs and carbon footprints with strategies that include procuring materials and services closer to the point of production or sale. Additionally, local sourcing often translates into faster lead times, allowing for greater responsiveness to market demands [29].
**IND-R15**:	Compliance with environmental regulations has become a pivotal aspect of modern industrial operations. Stricter standards on emissions, waste management, and resource utilization compel organizations to integrate sustainable practices at every stage of their value chain [65].
**IND-R16**:	Safety standards have evolved to encompass both human and machine interactions, leveraging advanced technologies like sensors, automation, and real-time monitoring to minimize risks. These comprehensive measures ensure the well-being of employees while maintaining high levels of productivity and operational efficiency. Integrated safety systems can detect hazardous conditions or human errors in real-time, enabling immediate corrective action [66].
**SOC-R1**:	One of the most significant instruments for transportation managers and engineers has been traffic surveillance and monitoring. Better congestion monitoring is a critical step in better understanding the reasons for the phenomena and facilitating more effective methods, particularly in complex multimodal contexts. Monitoring mobility movements from the air is becoming more common as technology improves and vision algorithms progress. This is a viable alternative to traditional monitoring techniques because data quality can be considerably improved and many types of transportation (private automobiles, public transportation, taxis, motorbikes, bikes, or pedestrians) can be examined [67].
**SOC-R2**:	In smart cities, public WiFi networks serve as the backbone for a plethora of interconnected systems, going beyond mere internet access for citizens. These networks facilitate real-time data exchange that drives innovations like autonomous public transport, smart grid management, and emergency services coordination. Public WiFi empowers community engagement, supports local businesses, and enhances urban living [68].
**SOC-R3**:	Telemedicine is revolutionizing healthcare by transcending geographical limitations and making medical consultations more accessible. Advanced algorithms and high-speed connectivity enable precise remote diagnoses, while wearable Internet of Things (IoT) devices continuously monitor vital signs, delivering real-time data to healthcare providers. This not only increases the efficiency of medical services but also enables personalized care, tailoring treatment to individual health metrics and histories [69].
**SOC-R4**:	Artificial intelligence (AI) developments have unleashed a wave of disruptive technologies, and one area that has seen tremendous success is AI-assisted diagnosis in healthcare. AI systems have the potential to change the diagnosis process as they enable earlier detection, increase accuracy, and individualize therapy recommendations by analyzing massive volumes of medical data, learning from patterns, and making correct predictions [70].
**SOC-R5**:	E-learning platforms are increasingly integral to modern education, offering personalized, accessible, and flexible learning experiences. Advanced analytics and adaptive algorithms tailor educational content to individual learning styles and paces, optimizing student engagement and retention. These platforms also democratize education by making high-quality resources available to anyone with internet access, breaking down traditional barriers like geography and socioeconomic status [71].
**SOC-R6**:	Lifelong learning initiatives are becoming increasingly vital in a rapidly evolving world, enabling individuals to adapt to new professional and societal demands. These programs, often delivered through online platforms, offer a broad array of courses that range from technical skills to emotional intelligence training. Lifelong learning entails more than just continuing formal education begun in schools. It requires making meaningful connections between learning and other aspects of daily life, such as work, family relationships, civic participation, and personal development [72].
**SOC-R7**:	Green buildings are increasingly seen as a cornerstone of sustainable urban development. Utilizing technologies like solar panels, energy-efficient appliances, and intelligent HVAC systems, these structures aim to minimize their ecological footprint. Advanced sensor networks monitor various parameters like temperature, air quality, and energy usage, optimizing building performance in real-time [73].
**SOC-R8**:	Renewable energy adoption is pivotal for addressing climate change and fostering a sustainable future. Advances in solar, wind, and hydro technologies are making these energy sources increasingly efficient and cost-competitive. Smart grids and battery storage solutions further enhance the viability of renewables by stabilizing supply and allowing for decentralized energy systems [74].
**SOC-R9**:	Income equality measures, such as progressive taxation and universal basic income, are gaining attention as mechanisms to redistribute wealth and opportunity more equitably. These policies aim to counteract the widening income gaps exacerbated by rapid technological advancements, ensuring that all citizens benefit from societal progress. Digital platforms and data analytics can help streamline the administration of such programs, making them more efficient and targeted [75].
**SOC-R10**:	Remote work technologies are reshaping the traditional office landscape, offering unprecedented flexibility and breaking down geographical barriers. Tools like video conferencing, virtual private networks, and collaborative software facilitate seamless communication and workflow management, enabling people to work from virtually anywhere. This decentralization of the workspace has positive implications for work-life balance and can even contribute to reduced carbon emissions due to less commuting [76].
**SOC-R11**:	Flexible scheduling is transforming the way work hours are perceived, offering a tailored approach that respects individual needs and lifestyles. This adaptability enables employees to maximize productivity and work during their peak hours thanks to digital tools and real-time analytics. It also supports a diverse workforce, accommodating those with caregiving responsibilities or educational commitments [77].
**SOC-R12**:	E-government services are streamlining administrative processes and enhancing citizen engagement. Digital platforms now offer everything from tax filing to license renewals, making these tasks more convenient and efficient. Blockchain and other secure technologies are increasingly being employed to ensure data integrity and safeguard personal information. These innovations not only reduce bureaucratic overhead but also make government more transparent and accountable [8].
**SOC-R13**:	Transparent policy-making is leveraging digital tools to foster an inclusive, participatory approach to governance. Platforms that enable real-time tracking of legislative processes, as well as online forums for public consultation, are making it easier for citizens to be informed and engaged. This openness enhances accountability and enriches decision-making through diverse inputs [78].
**SOC-R14**:	Data privacy has emerged as an important concern as digital interactions become increasingly central to daily life. Advanced encryption techniques and robust cybersecurity protocols aim to protect sensitive information from unauthorized access and breaches. Legislation like GDPR also plays a role, empowering individuals to have greater control over their personal data. However, the pervasive nature of data collection across various platforms, often without explicit user consent, raises ethical questions. Balancing the need for data to drive innovation with individual privacy rights remains a complex challenge that requires ongoing vigilance and dynamic policy frameworks [79].
**SOC-R15**:	Ethical AI is becoming a cornerstone of responsible technology deployment, aiming to ensure fairness, transparency, and inclusivity. Advanced algorithms are increasingly scrutinized for biases that may perpetuate social inequities or unfairly target specific groups. These ethical considerations are not just optional add-ons but integral to the development and implementation of AI systems [80].

The above-presented results of the qualitative analysis provide a significant insight into the complexity of Marketing 5.0, Industry 5.0, and Society 5.0.

## References

[1] Bakator M., Đorđević D., Ćoćkalo D. (2019). Modelling the influence of product development on business performance and competitiveness in manufacturing enterprises. Teh Vjesn.

[2] Mitić S., Popović J., Poštin J., Ćilerdžić V., Szabó L. (2021). Information technology as an indicator of the level of organizational performance. J Eng Manag Compet..

[3] Skobelev P.O., Borovik S.Y. (Industry 4.0. 2017). On the Way from Industry 4.0 to Industry 5.0: From Digital Manufacturing to Digital Society.

[4] Verma A., Bhattacharya P., Madhani N., Trivedi C., Bhushan B., Tanwar S., Sharma R. (2022). Blockchain for industry 5.0: vision, opportunities, key enablers, and future directions. IEEE Access.

[5] Sphinx I.T. (2019). https://www.sphinx-it.eu/fromthe-agenda-of-the-world-economic-forum-2019-society-5-0/.

[6] Keidanren. Society 5 (2018). 0-Co-creating the future. http://www.keidanren.or.jp/en/policy/2018/095_excerpt.pdf.

[7] Nakanishi H. (2019). Society 5.0 Will Liberate Us.

[8] Nikiforova A. (2021). Smarter open government data for society 5.0: are your open data smart enough?. Sensors.

[9] Assarkhaniki Z., Sabri S., Rajabifard A. (2021). Utilizing open data for the detection of informal settlement patterns: a step towards inclusive SDG achievement. Big Earth Data.

[10] Nikiforova A., Flores M.A.A., Lytras M.D. (2023). The role of open data in transforming the society to Society 5.0: a resource or a tool for SDG-compliant Smart Living?. Smart Cities Digit Transform.

[11] Alexa L., Pîslaru M., Avasilcăi S. (2022). Sustainability and Innovation in Manufacturing Enterprises: Indicators, Models and Assessment for Industry 5.0.

[12] Huang S., Wang B., Li X., Zheng P., Mourtzis D., Wang L. (2022). Industry 5.0 and society 5.0—comparison, complementation and co-evolution. J. Manuf. Syst..

[13] Martynov V.V., Shavaleeva D.N., Zaytseva A.A. (2019 Sep). 2019 Int Conf Qual Manag Transp Inf Secur Inf Technol (IT&QM&IS).

[14] Sołtysik-Piorunkiewicz A., Zdonek I. (2021). How society 5.0 and industry 4.0 ideas shape the open data performance expectancy. Sustainability.

[15] Paschek D., Luminosu C.T., Ocakci E. (2022). Sustainability and Innovation in Manufacturing Enterprises: Indicators, Models and Assessment for Industry 5.0.

[16] Trstenjak M., Mustapić M., Gregurić P., Opetuk T. (2023). Implementing green technologies in industry 5.0 for enhanced logistics. Tehnički glasnik.

[17] Mansurali A., Harish V., Ramakrishnan S. (2023). Transformation for Sustainable Business and Management Practices: Exploring the Spectrum of Industry 5.0.

[18] Kumar N., Sharma B., Narang S. (2021). Proceedings of Third International Conference on Computing, Communications, and Cyber-Security: IC4S.

[19] Sarıoğlu C.İ. (2023). Handbook of Research on Perspectives on Society and Technology Addiction.

[20] Kotler P., Kartajaya H., Setiawan I. (2021).

[21] Cheng Y., Jiang H. (2022). Customer–brand relationship in the era of artificial intelligence: understanding the role of chatbot marketing efforts. J. Prod. Brand Manag..

[22] Ganesan S., Gopalsamy S. (2022).

[23] Gilboa S., Seger-Guttmann T., Mimran O. (2019). The unique role of relationship marketing in small businesses' customer experience. J. Retailing Consum. Serv..

[24] Abdulhafedh A. (2021). Incorporating k-means, hierarchical clustering and pca in customer segmentation. J City Dev..

[25] Nguyen A.T., Parker L., Brennan L., Lockrey S. (2020). A consumer definition of eco-friendly packaging. J. Clean. Prod..

[26] Uzir M.U.H., Al Halbusi H., Thurasamy R., Hock R.L.T., Aljaberi M.A., Hasan N., Hamid M. (2021). The effects of service quality, perceived value and trust in home delivery service personnel on customer satisfaction: evidence from a developing country. J. Retailing Consum. Serv..

[27] Cvjetković M., Vasiljević M., Cvjetković M., Josimović M. (2021). Impact of quality on improvement of business performance and customer satisfaction. J Eng Manag Compet..

[28] Zizic M.C., Mladineo M., Gjeldum N., Celent L. (2022). From industry 4.0 towards industry 5.0: a review and analysis of paradigm shift for the people, organization and technology. Energies.

[29] Maderna R., Pozzi M., Zanchettin A.M., Rocco P., Prattichizzo D. (2022). Flexible scheduling and tactile communication for human–robot collaboration. Robot Comput-Integr Manuf..

[30] Aasma Nawang (2022 Aug). Digital marketing in the era of society 5.0 by applying design thinking. International Conference Faculty of Economics and Business.

[31] Kitchenham B. (2004).

[32] Kitchenham B., Brereton O.P., Budgen D., Turner M., Bailey J., Linkman S. (2009). Systematic literature reviews in software engineering–A systematic literature review. Inf. Software Technol..

[33] Moher D., Liberati A., Tetzlaff J., Altman D.G., Prisma Group (2010). Preferred reporting items for systematic reviews and meta-analyses: the PRISMA statement. Int. J. Surg..

[34] Page M.J., McKenzie J.E., Bossuyt P.M., Boutron I., Hoffmann T.C., Mulrow C.D. (2021). The PRISMA 2020 statement: an updated guideline for reporting systematic reviews. Bus. Manag. J.

[35] Sheth J. (2021). New areas of research in marketing strategy, consumer behavior, and marketing analytics: the future is bright. J Mark Theory Pract.

[36] Hair Jr JF., Sarstedt M. (2021). Data, measurement, and causal inferences in machine learning: opportunities and challenges for marketing. J Mark Theory Pract.

[37] Munusamy S., Murugesan P. (2020). Modified dynamic fuzzy c-means clustering algorithm–Application in dynamic customer segmentation. Appl. Intell..

[38] Laurie S., Mortimer K. (2019). How to achieve true integration: the impact of integrated marketing communication on the client/agency relationship. J Mark Manag.

[39] Rather R.A. (2020). Customer experience and engagement in tourism destinations: the experiential marketing perspective. J Travel Tour Mark.

[40] Følstad A., Skjuve M. (2019). Chatbots for customer service: user experience and motivation. Proc 1st Int Conf Conversat User Interfaces.

[41] Park Y., Mithas S. (2020). Organized complexity of digital business strategy: a configurational perspective. MIS Q..

[42] Chen Y., Kwilinski A., Chygryn O., Lyulyov O., Pimonenko T. (2021). The green competitiveness of enterprises: justifying the quality criteria of digital marketing communication channels. Sustain. Times.

[43] Wang C.L. (2021). New frontiers and future directions in interactive marketing: inaugural Editorial. J Res Interact Mark.

[44] Pahlevi M.R., Suhartanto D. (2020). The integrated model of green loyalty: evidence from eco-friendly plastic products. J. Clean. Prod..

[45] Okazaki S., Plangger K., West D., Menéndez H.D. (2020). Exploring digital corporate social responsibility communications on Twitter. J. Bus. Res..

[46] Wu X., Li Y., Zhu Z. (2023). Does online–offline channel integration matter for supply chain resilience? The moderating role of environmental uncertainty. Ind. Manag. Data Syst..

[47] Palazón M., López M., Sicilia M., López I. (2022). The customer journey: a proposal of indicators to evaluate integration and customer orientation. J Mark Commun..

[48] Ho J., Pang C., Choy C. (2020). Content marketing capability building: a conceptual framework. J Res Interact Mark..

[49] Müller J., Christandl F. (2019). Content is king–But who is the king of kings? The effect of content marketing, sponsored content & user-generated content on brand responses. Comput. Hum. Behav..

[50] Cambier F., Poncin I. (2020). Inferring brand integrity from marketing communications: the effects of brand transparency signals in a consumer empowerment context. J. Bus. Res..

[51] Jung (2023). Research trends of sustainability and marketing research, 2010–2020: topic modeling analysis. Heliyon.

[52] Fatima Z., Tanveer M.H., Waseemullah Zardari S., Naz L.F., Khadim H., Tahir M. (2022). Production plant and warehouse automation with IoT and industry 5.0. Appl. Sci..

[53] Frick J., Grudowski P. (2023).

[54] Jabrane K., Bousmah M. (2021). A new approach for training cobots from small amount of data in industry 5.0. Int. J. Adv. Comput. Sci. Appl..

[55] Leng J., Sha W., Wang B., Zheng P., Zhuang C., Liu Q., Wang L. (2022). Industry 5.0: prospect and retrospect. J. Manuf. Syst..

[56] Moraes A., Carvalho A.M., Sampaio P. (2023). Lean and industry 4.0: a review of the relationship, its limitations, and the path ahead with industry 5.0. Machines.

[57] Maddikunta P.K.R., Pham Q.V., Prabadevi B., Deepa N., Dev K., Gadekallu T.R., Liyanage M. (2022). Industry 5.0: a survey on enabling technologies and potential applications. J Ind Inf Integr.

[58] Li X., Zhang P., Chen G., Wang W., Li J. (2020). Waste minimization and efficient disposal of particles in optimized organic silicon production. J. Clean. Prod..

[59] Saptaningtyas W.W.E., Rahayu D.K. (2020 Mar). Proc Int Conf Ind Eng Oper Manag.

[60] van Oudenhoven B., Van de Calseyde P., Basten R., Demerouti E. (2022). Predictive maintenance for industry 5.0: behavioural inquiries from a work system perspective. Int. J. Prod. Res..

[61] Javeed D., Gao T., Kumar P., Jolfaei A. (2023). An explainable and resilient intrusion detection system for industry 5.0. IEEE Trans. Consum. Electron..

[62] Ghobakhloo M., Iranmanesh M., Mubarak M.F., Mubarik M., Rejeb A., Nilashi M. (2022). Identifying industry 5.0 contributions to sustainable development: a strategy roadmap for delivering sustainability values. Sustain. Prod. Consum..

[63] Pereira R., dos Santos N. (2023). Neoindustrialization—reflections on a new paradigmatic approach for the industry: a scoping review on industry 5.0. Logistics.

[64] Adel A. (2022). Future of industry 5.0 in society: human-centric solutions, challenges and prospective research areas. J. Cloud Comput..

[65] Guruswamy S., Pojić M., Subramanian J., Mastilović J., Sarang S., Subbanagounder A., Jeoti V. (2022). Toward better food security using concepts from industry 5.0. Sensors.

[66] Barmpounakis E., Geroliminis N. (2020). On the new era of urban traffic monitoring with massive drone data: the pNEUMA large-scale field experiment. Transp Res Part C Emerg Technol.

[67] Raju R., Abd Rahman N.H., Ahmad A. (2022). Cyber security awareness in using digital platforms among students in a higher learning institution. Asian J Univ Educ.

[68] Dorsey E., Okun M.S., Bloem B.R. (2020). Care, convenience, comfort, confidentiality, and contagion: the 5 C's that will shape the future of telemedicine. J. Parkinsons Dis..

[69] Wang J. (2023). The power of AI-assisted diagnosis. EAI Endorsed Trans e-Learning..

[70] Dahan N.A., Al-Razgan M., Al-Laith A., Alsoufi M.A., Al-Asaly M.S., Alfakih T. (2022). Metaverse framework: a case study on E-learning environment (ELEM). Electron.

[71] Hirju I., Georgescu R.I. (2023). The concept of learning cities: supporting lifelong learning through the use of smart tools. Smart Cities.

[72] Qiang G., Tang S., Hao J., Di Sarno L., Wu G., Ren S. (2023). Building automation systems for energy and comfort management in green buildings: a critical review and future directions. Renew. Sustain. Energy Rev..

[73] Eshchanov B., Abdurazzakova D., Yuldashev O., Salahodjaev R., Ahrorov F., Komilov A., Eshchanov R. (2021). Is there a link between cognitive abilities and renewable energy adoption: evidence from Uzbekistan using micro data. Renew. Sustain. Energy Rev..

[74] Tang C.K., Macchia L., Powdthavee N. (2023). Income is more protective against pain in more equal countries. Soc. Sci. Med..

[75] García G.D., Calvache C.J.P., Rodríguez F.J.Á. (2022). Society 5.0 and soft skills in agile global software development. IEEE Rev Iberoam Tecnol Aprendiz.

[76] Zhang R., Lv J., Bao J., Zheng Y. (2023). A digital twin-driven flexible scheduling method in a human–machine collaborative workshop based on hierarchical reinforcement learning. Flex. Serv. Manuf. J..

[77] Aminah S., Saksono H. (2021). Digital transformation of the government: a case study in Indonesia. J Komun: Malaysian J Commun.

[78] Amo Filvá D., Prinsloo P., Alier Forment M., Fonseca Escudero D., Torres Kompen R., Canaleta Llampallas X., Herrero Martín J. (2021). Local technology to enhance data privacy and security in educational technology. Int J Interact Multimed Artif Intell..

[79] Zhang X., Wei X., Ou C.X., Caron E., Zhu H., Xiong H. (2022). From human-AI confrontation to human-AI symbiosis in society 5.0: transformation challenges and mechanisms. IT Prof.

[80] Bibri S.E., Alexandre A., Sharifi A. (2023). Environmentally sustainable smart cities: converging AI, IoT, and big data technologies and solutions. Energy Inform.

[81] Alojail M., Khan S.B. (2023). The impact of digital transformation on sustainable development. Sustainability.

[82] United Nations Development Programme (2022). Digital strategy 2022-2025. https://digitalstrategy.undp.org/.

[83] Sima E. (2021).

[84] Dutt V. (2023). Marketing 5.0: the era of technology and challenges. International Journal of Advances in Engineering and Management.

[85] Gladden M.E. (2019). Who will Be the members of society 5.0? Towards an anthropology of technologically posthumanized future societies. Soc. Sci..

[86] Aquilani B., Piccarozzi M., Abbate T., Codini A. (2020). From industry 4.0 to society 5.0: open innovation and value Co-creation. Sustainability.

[87] MacPherson J., Voglhuber-Slavinsky A., Olbrisch M., Schöbel P., Dönitz E., Mouratiadou I., Helming K. (2022). Future agricultural systems: digitalization for sustainability goals. Agron. Sustain. Dev..

[88] Kulkov I., Kulkova J., Rohrbeck R., Menvielle L., Kaartemo V., Makkonen H. (2023). AI-Driven sustainable development: organizational, technical, and processing approaches. Sustain. Dev..

[89] Alojaiman B. (2023). Technological modernizations in industry 5.0 era: descriptive analysis and future research directions. Processes.

[90] Lechevalier S. (2024). Society 5.0 and new capitalism: complementarities and contradictions. Asia Pac. Bus. Rev..

[91] Slavic D., Marjanovic U., Medic N., Simeunovic N., Rakic S. (2024). The evaluation of Industry 5.0 concepts: social network analysis approach. Appl. Sci..

[92] Bakator M., Nikolić M., Ćoćkalo D., Stanisavljev S. (2024). Transition to Industry 5.0 with AI and digitalization of production systems. J Eng Manag.

[93] Movahed A.B., Movahed A.B., Nozari H. (2024). Opportunities and challenges of marketing 5.0. Smart Sustain Interact Mark.

[94] Zulfitri Z. (2024). Antecedents of customer loyalty to increase banking market share in the Marketing 5.0 era. ADPEBI Int J Bus Soc Sci.

[95] Kumar V., Kotler P. (2024). Transformative Marketing: Combining New Age Technologies and Human Insights.

[96] Choudhry M.D., Jeevanandham S., Sundarrajan M., Jothi A., Prashanthini K., Saravanan V. (2024).

[97] Ziatdinov R., Atteraya M.S., Nabiyev R. (2024). The fifth industrial revolution as a transformative step towards Society 5.0. Societies.

